# Advanced separation and classification of ionospheric troughs in midnight conditions

**DOI:** 10.1038/s41598-022-17591-4

**Published:** 2022-08-04

**Authors:** Alexander Karpachev

**Affiliations:** Pushkov Institute of Terrestrial Magnetism, Ionosphere and Radio Wave Propagation (IZMIRAN), Moscow, Russia

**Keywords:** Space physics, Astronomy and planetary science

## Abstract

This study uses a novel approach to separate and classify different ionospheric troughs from CHAMP satellite data in the winter midnight ionosphere of the southern hemisphere at high solar activity (2000–2002). The main ionospheric trough (MIT) was separated from the high latitude trough (HLT). The separation was performed through an analysis of troughs in the frame of a model of the diffuse auroral particle precipitation. Two types of HLT were distinguished. In the mid-latitude ionosphere, the MIT was separated from the ring ionospheric trough (RIT), which is formed by the decay processes of the magnetospheric ring current. The separation was performed on the basis of an analysis of the prehistory of all geomagnetic disturbances for the period under study. In addition, a decrease in the electron density, which is superimposed on the MIT and masks its minimum position, is quite often observed at American and Atlantic longitudes near the Polar Circle.

## Introduction

The ionization trough was discovered from the Alouette I satellite data and was described by Muldrew^1^ as the main ionospheric trough (MIT). Since then, many studies have explored its characteristics, which have been described in reviews^2–5^. The greatest attention has been paid to the position of the MIT minimum^2,6–13^. These studies reported a large data scatter, which is due to the fact that the MIT can be confused with other troughs, including high latitude troughs (HLT) and low latitude troughs^9,14,15^. Thus, the problem of the separation and classification of ionospheric troughs arises. Significant progress in the separation of the MIT and HLT has been made in a previous study based on CHAMP data^15^. The MIT is a subauroral structure because it is located equatorward of the auroral oval^4^. The HLT is observed inside the auroral oval^16^. With the simultaneous data on the precipitation of auroral particles, the separation of the MIT and HLT would be a routine task. However, particle precipitation on board the CHAMP has not been measured simultaneously; therefore, we were forced to use a statistical model of diffuse auroral precipitation. Thus, the current positions of the troughs have been compared with some average positions of the equatorward boundary of the auroral oval^15^. However, the current positions of the auroral oval and the troughs are often quite different from the average position. For example, the standard deviation for the MIT position is typically 2°–3°, and the maximal data scatter is as high as ± 10°^6,9,12^. As a result, the highest latitude MIT case can be located inside the statistical auroral oval, while the lowest latitude HLT case can be outside it.

In the present study, an advanced method is used. The key point is an application of a model of auroral precipitation obtained from the DMSP satellites data^17,18^. This model describes the position of zone I diffuse precipitation at the equatorward edge of the auroral oval and zone II at its poleward edge. As is known, the precipitation in zone I forms the poleward wall of the MIT; meanwhile, the effects of zone II have never been considered. Moreover, the positions of both zones change with longitude^18,19^. Therefore, for superior efficiency, the analysis of the structures of the high latitude ionosphere was conducted herein in the framework of the longitudinal effect.

The problem of low-latitude trough separating from the subauroral MIT in the previous study was solved by removing from the CHAMP data set only the obvious cases of the so-called ring ionospheric trough (RIT)^15^. The RIT is formed during the storm (substorm) recovery phase as a result of the decay of the magnetospheric ring current^20,21^. However, equatorward of the MIT, in addition to the mid-latitude RIT, other electron density minima do not necessarily stand out as ionization troughs, but they significantly complicate the identification of the MIT. Therefore, this study also considered in detail the issue of the separation of the MIT and LLT.

Finally, to complete the pattern, this study highlights the cases of a clearly expressed polar hole. In this way, the title of this article can be interpreted broadly as the classification of electron density depletion structures in the high and mid-latitude ionosphere. Within the framework of the advanced method, all the ionospheric troughs from the CHAMP data in the midnight winter ionosphere were thoroughly analyzed. As the main goal was to derive an accurate statement of the problem for the trough separation, the analysis in the present study was limited to the southern hemisphere and high solar activity.

## Observation data

The CHAMP satellite carried out in situ measurements of electron concentration *Ne*^22^*.* Variations in *Ne* are presented below in terms of plasma frequency *fp* (*Ne*[cm^–3^] = 1.24 × 10^4^*fp*^2^[MHz]). The CHAMP altitude has changed from ~ 450 to ~ 300 km, which is close to the height of the F2 layer maximum. It revolved on nearly polar orbit with the inclination of 87°. The CHAMP data time resolution of 15 s is less than 1° of latitude, which allows determining the minimum trough position accurately. The CHAMP data are available on the website http://op.gfz-potsdam.de/champ.

The CHAMP data for June, July, and August (i.e., for local winter conditions) in the southern hemisphere were used. The data only for high solar activity with F10.7 ~ 180 sfu for the period of 2000–2002 and the near-midnight conditions (23–01 LT) were considered. About 700 CHAMP passes in the winter high- and mid-latitude ionosphere for relatively quiet geomagnetic conditions with Kp ≤ 4 were examined.

The MIT is usually defined by a fairly deep decrease in electron density of at least ~ 30%. We have not determined the level of electron density decrease in the MIT minimum. If the trough was poorly expressed on some satellite path or masked by ionospheric plasma irregularities, then the position of its minimum was determined through coordination with neighboring paths. Stricter criteria were imposed on the selection of the HLT. The HLT is observed in the auroral oval, where the electron density is highly irregular and a number of density minima can be observed. Therefore, the HLT was recorded only in the obvious cases wherein it was clearly structured and when its poleward wall did not extend beyond the poleward diffuse precipitation zone. Similarly, the polar hole was defined only as a broad minimum of the electron density at latitudes above the poleward precipitation zone. Finally, only pronounced troughs were recorded equatorward of the MIT.

## Structure of nighttime ionosphere

An analysis of the structures of the high latitude ionosphere was conducted using a model of auroral particle precipitation constructed from the DMSP satellites data in both hemispheres^17,18^. The model is uploaded on the website of the Polar Geophysical Institute (http://apm.pgia.ru). In Fig. S1 (in “Supporting information”), this model is presented for quiet conditions. The model describes three main auroral precipitation zones: diffuse auroral zone I equatorward of the auroral oval, structured auroral precipitation of the auroral oval (region of auroral lights, aurora), and zone II of the soft diffuse precipitation poleward of the aurora.

**Figure 1:**
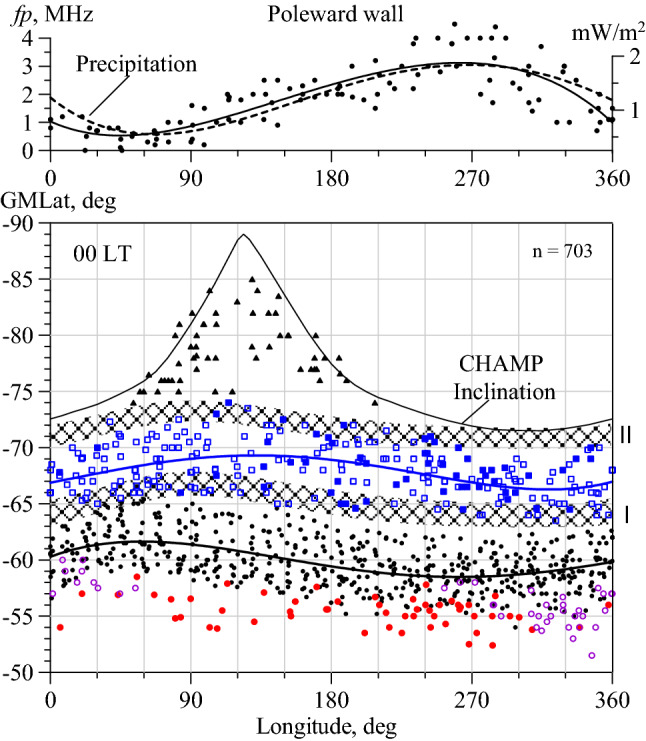
On the top: Longitudinal variations in the magnitude of MIT poleward wall (dots and approximation) and averaged auroral precipitation energy flux at 21-03 MLT under Kp = 2 (dashed line)^19^. On the bottom: Longitudinal variations in the positions of main structures in the near-midnight winter ionosphere of the southern hemisphere: polar hole (triangles), HLT1 (empty squares), HLT2 (filled squares), MIT (black dots), RIT (red dots), and specific electron density minima (purple empty circles). The shaded latitude belts show the diffuse auroral precipitation in zones I and II^19^. The upper curve represents the CHAMP inclination equal to 87°.

The boundaries of the precipitation zones in the midnight ionosphere change with longitude^18,19^, as well as the position of the MIT^23^. In the southern hemisphere these boundaries were revealed from the TIMED data obtained in 2002–2007^19^. They are presented in Fig. S2 (in “Supporting information”). The equatorward and poleward boundaries of the oval experience synchronous longitudinal variations with an amplitude of ~ 2.5°. Therefore, it is most effective to analyze the structures of the high latitude ionosphere in terms of geomagnetic latitude–geographic longitude. Figure 1 (bottom panel) shows the positions of the different structures in the winter midnight (23-01 LT) ionosphere of the southern hemisphere. To eliminate the dependence on geomagnetic activity, the positions of the MIT, RIT, and HLT were reduced to Kp = 2 according to Λcorr = Λc − *a*(Kp(τ) − 2), where Λc is the current position of the structure and the ***a*** factor is 2.0° for the MIT^8^, 1.5° for RIT^21^, and ~ 1.5° for HLT^16^. The Kp(τ) index was used as it considers the prehistory of geomagnetic activity development^11^. In Fig. 1, zones I and II of the diffuse precipitation taken from Fig. S1 are shaded. The average (for all longitudes) position of the equatorward boundary of the auroral precipitation oval corresponds to 64° at Kp = 2^15^. The upper curve in Fig. 1 (bottom panel) corresponds to the CHAMP satellite inclination. The satellite inclination of 87° does not limit the observations of the discussed structures, except for the polar hole. But polar hole cases are shown in Fig. 1 solely for the completeness of the pattern; only unambiguous cases were selected.

**Figure 2:**
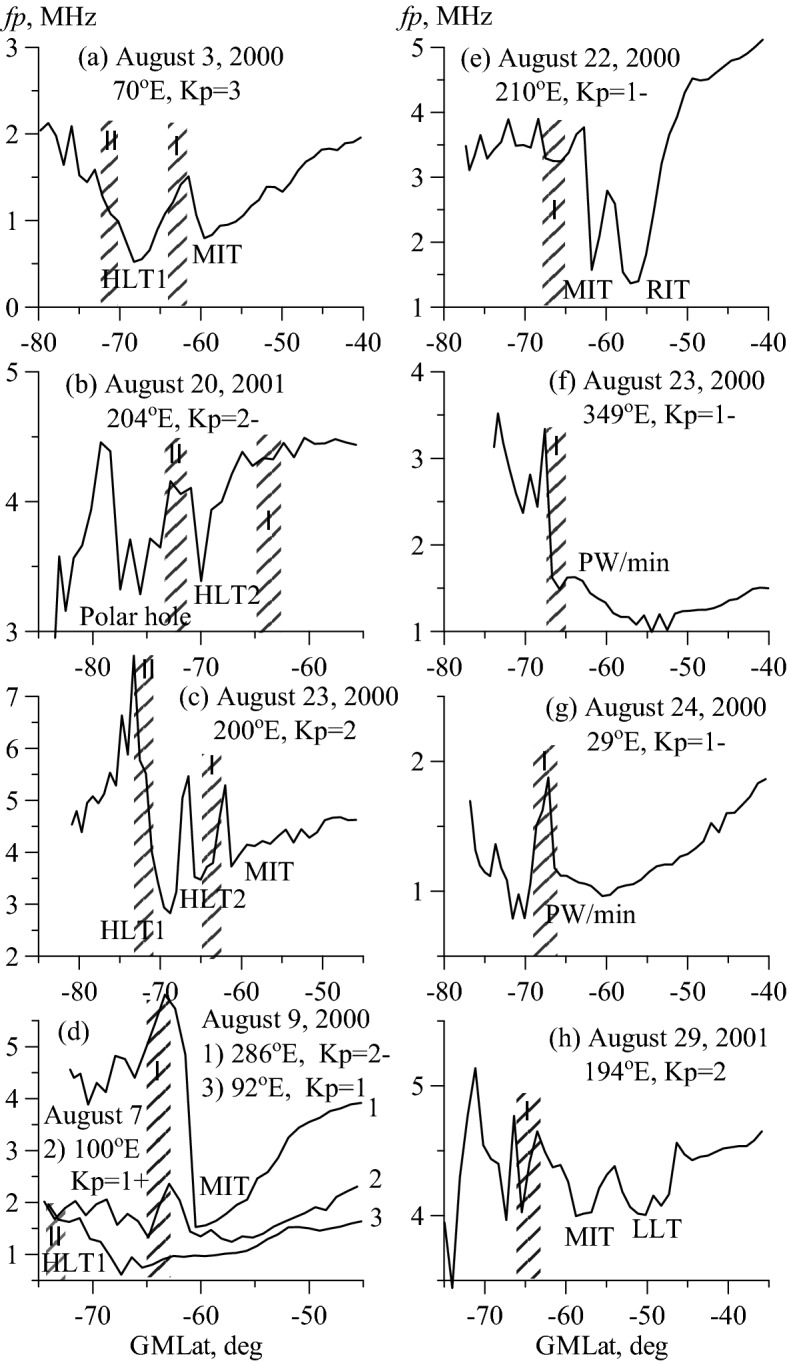
The most characteristic examples of troughs in the nighttime winter ionosphere of the southern hemisphere. Local time changes from 23.8 to 0.9 h. The hatching bars show the zones I and II of precipitation taking into account longitude and Kp index. Details are in the text.

The black dots in Fig. 1 depict the cases of MIT observations (n = 703). The approximating curve demonstrates the longitudinal effect in the MIT position with an amplitude of ~ 3° and a correlation coefficient of 0.52. The data scatter (standard deviation) is 1.85°, which is less than the 2°–3° value that is usually observed in the statistical processing of the trough data. In the first approximation, the longitudinal variations in the MIT position are consistent with the variations in the position of the precipitation in zone I. The main task was to separate the MIT from HLT (blue squares) at the high latitude boundary of the MIT occurrence region. Figure 2a shows the simplest case when both troughs are observed simultaneously. This case allows us to draw a fundamentally important conclusion: the MIT poleward wall is, as usual, determined by the precipitation in zone I, and the HLT poleward wall is undoubtedly formed by the precipitation in zone II. The latter fact is the key to the identification of the HLT1. The HLT was previously studied in detail from the *Ni* variations recorded on board OGO-6 at heights of 400–1100 km^16^ and from EISCAT radar data^24^. In particular, the statistical position of HLT relative to the auroral oval was determined^16^. The authors observed the HLT exclusively within the auroral oval and attributed its formation ultimately to the action of electric fields in the zone of the convection of the high latitude ionospheric plasma. These fields cause the frictional heating and upward vertical drift of the plasma. The first process leads to an increase in recombination, the second one to the escape of plasma upward along the magnetic field lines. Since this effect is observed in a limited region, the HLT of this type is usually narrow (3°–5° in latitude). Such a trough is observed in Fig. 2b together with the polar hole. We define such a trough as the HLT2; it is depicted by filled squares in Fig. 1. Figure 2c shows a rather rare example of the simultaneous observation of the three troughs: MIT, HLT2, and HLT1. Figure 1 shows that HLT2 is observed less frequently than HLT1. In Fig. 2, an approximation curve for all high latitude troughs (HLT1 and HLT2) is drawn. For the visibility, the precipitation zones are shown by hatching in Fig. 2. They are located considering the longitude and the Kp index value. However, it should be remembered that the precipitation zones are taken from the model and may not exactly correspond to the trough current position.

In the eastern hemisphere, at longitudes of 30°–90° E, the MIT is located at the highest latitudes so that the region of its existence overlaps with the precipitation in zone I and the region of HLT existence. In the region of the intersection of the two sets of troughs, the problem of separation becomes particularly acute. Therefore, all cases of trough observations in this region were analyzed thoroughly. The top panel in Fig. 1 shows the longitudinal variations in the magnitude of the poleward wall (PW) derived from the CHAMP data for the quiet period of August 15–24, 2000 (dots and approximation line). The longitudinal effect is detected confidently, which is quite surprising, bearing in mind the extremely irregular character of diffuse precipitation. The dashed line depicts the longitudinal variations in the average precipitation energy flux derived at latitude of − 65° GMLat from the colored Fig. S2^19^. As one might expect, the variations in the magnitude of the PW completely coincide with variations in the precipitation. However, the high degree of coincidence is also surprising. Electron precipitation is much stronger in the western hemisphere than in the eastern hemisphere. Therefore, in the western hemisphere, the precipitation forms a pronounced PW of the MIT, which is always clearly determined. This illustrates the latitudinal *fp* cross-section in Fig. 2d, which represents the MIT recorded on August 9, 2000, at longitude of 286° E at 0.6 LT and Kp = 2−. In the eastern hemisphere at problematic longitudes different scenarios can be realized. If the precipitation in zones I and II is still quite intense, they form (weak) peaks of electron density, and both troughs are observed. If the precipitation in one of the zones is very weak, then either the MIT or the HLT can be formed. For example, curve 2 in Fig. 2d represents the latitudinal *fp* cross-section obtained on August 7, 2000, at longitude of 100° E at 0.5 LT and Kp = 1+. The latitudinal profile 2 shows weak electron density peak at the same latitudes as profile 1, i.e. at latitudes of zone I of precipitation. Hence, we can talk about the formation of weakly expressed MIT. The latitudinal profile 3 was also recorded on August 9, 2000, but at longitude of 92° E. Here is neither a peak nor a minimum of electron density at the latitudes of the MIT, therefore the MIT is not identified in this case. The minimum of the electron density is observed much poleward at latitude of − 68°, and it certainly belongs to HLT1 because its PW is formed by the precipitation in zone II. Note that this trough can be easily confused with the MIT in a cursory analysis. Finally, if both zones have no precipitation, then a monotonous decrease is recorded in the electron density to the pole without peaks and troughs. Such cases correspond to the *fp* values close to 0 on the top panel in Fig. 1.

**Figure 3:**
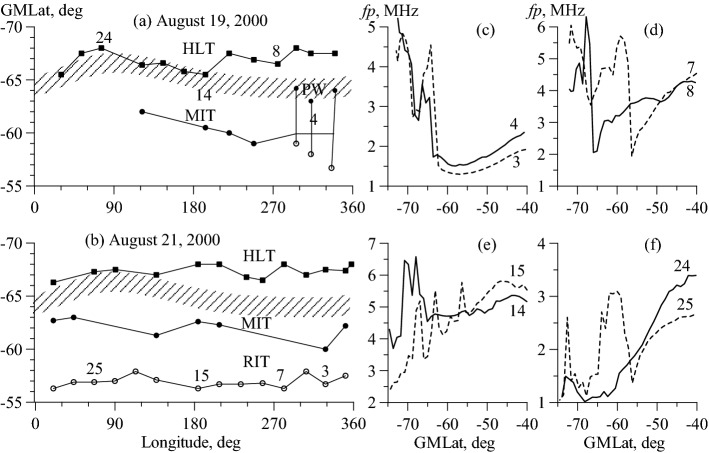
Longitudinal variations in the position of HLT (squares) and MIT (dots) on August 19 2000 (**a**) and August 21, 2000 (**b**). Empty circles show the positions of the *fp* minima (top) and RIT (bottom). The shaded latitude belts show the diffuse auroral precipitation in zone I. On the right are the latitudinal profiles of *fp* for the paths marked in the Figure on the left (solid curves for August 19, dashed curves for August 21).

The red dots in Fig. 1 depict the RIT cases that were observed equatorward of the MIT. The RIT forms during the recovery phase of a geomagnetic storm and even a weak substorm because of the decay of the magnetospheric ring current. The dynamics of this mid-latitude trough was described in detail earlier^20,21^. When the MIT and RIT are simultaneously observed, their identification is not difficult; the MIT position corresponds to the model^8^ and precipitation in zone I, at that time, the equatorward trough is the RIT (Fig. 2e). However, during a storm, any situation can be observed: both troughs, one MIT, or one RIT. Moreover, the MIT can be identified on one path, and the RIT on the next path. Therefore, the main method of MIT and RIT separation is an analysis of the prehistory of geomagnetic disturbance development^20,21^. Herein, even weak geomagnetic disturbances for the period under consideration were analyzed to separate the RIT from the MIT. An example of such an analysis is applied below in the discussion of Fig. 3.

Figures 2f,g show examples of structures that can be defined as quasi-troughs. Figure 2fshows the latitudinal *fp* cross-section typical for the longitudes of America and the Atlantic: steep poleward wall (PW) of the trough, shallow electron density minimum slightly equatorward (at − 65.5°), and deep and wide minimum at − 55°. How is the position of the MIT determined in this case? The latitude of − 65.5° for Kp = 1− corresponds rather to the PW of the MIT, and the latitude of − 55° completely goes beyond the existence region of “normal” MIT. Similarly, the position of *Ne* minimum at latitude of − 60.5° for Kp = 1− in Fig. 2g is definitely lower than the “normal” position of the MIT at longitude of 29° E (Fig. 1). The well-defined PW of the trough allows us to solve this problem. In the midnight hours, the base of the PW usually coincides with the equatorward boundary of diffuse precipitation^25^. The MIT minimum is located within 5° equatorward of this boundary^4^, and the minimum distance is about 2°^26^; therefore, the MIT minimum is usually 3°–4° equatorward of the PW. The minimum of the trough determined in this way in Fig. 2f,g coincides with the average position of the MIT (Fig. 1). As for the reason for the formation of an additional minimum of electron density, we should note that the geomagnetic latitude of − 56° at longitude of 285° approximately corresponds to the geographical latitude of − 66°, that is, the Polar Circle. The Polar Circle limits the area of the polar night in winter conditions, wherein there is no solar ionization and the electron density decays. The influence of the polar night affects a fairly wide range of longitudes from 120° W to 30° E.

Finally, Fig. 2h shows an example of a clearly defined minimum of electron density recorded on August 29, 2001, at latitude of − 50.2° and longitude 194° E. Several well-expressed LLTs were observed at latitudes − 50° and equatorward (not shown in Fig. 1). They apparently belong to the class of LLTs discovered earlier^27^.

## Events on August 19 and 21, 2000

The trough identification is obviously a challenge. In some cases, data analysis turns into a complicated investigation. Figure 3 shows two examples that required such thorough investigation. Figure 3 shows the longitudinal variations in ionospheric structures for August 19 (a) and August 21 (b), 2000. The data were obtained in the near-midnight sector for the average value of Kp = 1 on August 19 and Kp = 2+ on August 21. However, at the beginning of August 21, the Kp index increased from a value of 1 to 3+, and this change was enough to form deep RIT (circles in Fig. 3b), which was then observed all day in a pronounced form at latitudes of 56°‒58°. The RIT examples are observed on satellite paths 7, 15, and 25 in Fig. 3 on the right. The MIT identification was a challenge. Its position was confidently defined only on paths 3 and 15. Conversely, the HLT was clearly revealed all day, as shown by the paths 3, 7, 15, and 25 on the right of Fig. 3. Under very quiet conditions on August 19, the RIT did not manifest itself.

The vertical lines in Fig. 3a combine the structures represented by profile 4 in Fig. 3c. It consists of a shallow Ne minimum at the base of the MIT poleward wall and a rather deep equatorward minimum (red circles). The base of the PW corresponds rather to the zone I of precipitation, and the second minimum is formed much more equatorward than the average position of the "normal" MIT, especially on path 2. This structure has already been discussed in the description of Fig. 2f,g. Here is a problem of determining the exact position of the MIT minimum. In Fig. 3, the position of the MIT on paths 2‒6 is determined according to the approximation in Fig. 1. The rest of the time, the MIT manifested itself at best in the form of a shallow electron density minimum, as observed on paths 14 and 24. The HLT was also clearly manifested, particularly on paths 4, 14, and 24. On path 8, only the HLT was observed. Therefore, in both cases, there was well-expressed HLT, and on August 21, the RIT was also well-expressed. Much effort was needed to distinguish the MIT in both cases. Moreover, on path 8 on August 19, under manual and automatic data processing, the HLT would have been identified as the MIT.

## Conclusion

Undoubtedly, considerable progress has been made in the separation and classification of the various structures of the nighttime high latitude and mid-latitude ionosphere. The success is based on several factors. First, the CHAMP large data set allows the consideration of a phenomenon from different angles. Second, all complex cases were analyzed carefully, and automatic data processing was found to be questionable. Third, the problem could only be solved after many years of experience. Note that the solution to the problem could be traced back to 1997‒1998^9,14^, and it was continued in 2019^15^. Fourth, the idea of separating MIT, HLT1, and HLT2 arose from a simple and illustrative model of diffuse auroral precipitation^17^. It describes precipitation in zone I on the equatorward edge of the auroral oval and in zone II on its poleward edge. It turned out that precipitation in zone II forms the PW of HLT1, similar to the way the precipitation in zone I forms the PW of the MIT. This point is key in the separation of MIT and HLT1.

As the boundaries of both zones change with longitude by 2.5°^19^, similar to the longitudinal variations in the MIT position, the analysis is most effective when performed in the framework of the longitudinal effect. The problem of the separation of MIT and HLT1 was found to be radically different in the western and eastern hemispheres. In the western hemisphere, the MIT is located at lower latitudes than in the eastern hemisphere and is equatorward of the auroral oval. In the western hemisphere, the intensive precipitation forms a very steep and high PW of the MIT. These conditions facilitate the separation of MIT and HLT. In the eastern hemisphere, MIT shifts to high latitudes so that the region of its existence at longitudes 30°‒90° E overlaps zone I of the precipitation and region of HLT existence. In addition, the weak precipitation at longitudes of 0°‒90° E produces much less expressed and irregular electron density structures. Therefore, at these longitudes, each case was considered especially carefully, and the separation of MIT and HLT1 was carried out according to the correspondence of the PW to the precipitation in zone I or II. The pattern is complicated by the presence of a second high latitude trough (HLT2) described in^16,24^. Fortunately, HLT2 differs in that it is relatively narrow in latitude (3°‒4°).

The mid-latitude troughs (RITs) and sub-troughs (Ne minimums) located equatorward of the MIT were also clearly separated from the MIT for the first time. The main one among them is the RIT. It is formed even after a weak enhancement of geomagnetic activity, and it can be observed for a long time (sometimes for two days) at latitudes near L ~ 3^20,21^. It is no less difficult to separate the MIT from the RIT than the MIT from the HLT, but the methodology for such a separation has been carefully developed earlier^20,21^. It is based mainly on the prehistory of the disturbance development. Therefore, even the weak geomagnetic disturbances during the period under consideration were carefully analyzed. Note that RITs are more often formed at longitudes with a weak geomagnetic field, i.e., in the western hemisphere.

The quasi-trough is understood as an additional minimum of the electron density equatorward of the MIT, and it is often observed at longitudes of America and Atlantic. It is assumed to be related to the decay of the electron density beyond the Polar Circle during the polar night. This minimum deepens the MIT and therefore prevents the determination of the exact position of the MIT. Finally, several troughs too far from the mean MIT position (< 50°) were recorded, and they, apparently, belong to LLTs^27^.

The result of this analysis is a more accurate determination of the MIT position: the standard deviation of 1.85° is less than that in other statistical studies, and the scatter has decreased to ± 4°. This allows to significantly refine the model of the MIT position.

The study considers the structure of the ionosphere for limited conditions: high solar activity, winter, southern hemisphere, and near-midnight conditions. Preliminary analysis shows that the structure of evening and morning ionosphere is quite different from the considered structure. The same is particularly true for the daytime ionosphere. Consequently, this work should be considered as a statement of the problem, which implies the need for further research.

## Supplementary Information

Supplementary Information.
