# Supplementary material for: Advanced separation and classification of ionospheric troughs in midnight conditions
Source: Sci Rep. 2022 Aug 4;12:13434. doi: 10.1038/s41598-022-17591-4 (PMC9352663; doi:10.1038/s41598-022-17591-4)
Supplement: Supplementary file 1 — Supplementary Information. [file 41598_2022_17591_MOESM1_ESM.docx]

Supporting Information for

**Advanced separation and classification of ionospheric troughs in midnight conditions**

Karpachev A.T.

**The model of the auroral oval of precipitation**

The model of auroral particle precipitation is based on data from a large set of DMSP satellites in the Northern and Southern hemispheres [Vorobjev and Yagodkina, 2005, 2010]. The model is uploaded on the website of the Polar Geophysical Institute: http://apm.pgia.ru. Figure S1 shows this model for quiet conditions AL = -10 nT, Dst = -5 nT (Kp~2). As can be seen from Fig.S1, the model describes three main zones of auroral particle precipitation**.**

Figure S1. Model of auroral particle precipitation: diffuse auroral zone I equatorward of aurora (blue), structured auroral oval precipitation (auroral lights region or aurora, green), and soft diffuse precipitation zone II (orange) poleward of aurora.


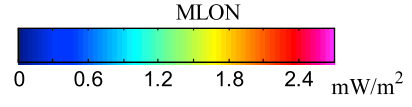


Figure S2. Longitudinal variations in the averaged auroral precipitation energy flux at 21 – 03 MLT under Kp = 2 for the June solstice (Jun.) in southern hemisphere [Luan et al., 2011].

Note, that plot is presented in geomagnetic longitude MLON.
